# Clinical management, economic and quality-of-life impacts among consulting people with obesity in Brazil: results from a real-world survey

**DOI:** 10.20945/2359-4292-2025-0155

**Published:** 2025-09-28

**Authors:** Priscila S. Barroso, Andrea Leith, Lewis Harrison, Fabiana M. Cyrulnik, Esther Artime, Gustavo Akerman Augusto

**Affiliations:** 1 Eli Lilly do Brasil, São Paulo, SP, Brazil; 2 Adelphi Real World, Bollington, United Kingdom; 3 Eli Lilly Spain, Alcobendas, Madrid, Spain; 4 CPQuali Pesquisa Clínica, São Paulo, SP, Brazil

**Keywords:** Brazil, obesity management, cost of illness, quality of life, burden, cross-sectional study

## Abstract

**Objective:**

Obesity prevalence is increasing in Brazil. Real-world observational data were used to understand clinical weight management practice, and the economic and health-related quality-of-life (HRQoL) impact of obesity.

**Materials and methods:**

Data were derived from the Adelphi Real World Obesity Disease Specific Programme (DSP)™, a cross-sectional survey of people with obesity (PwO) and treating physicians, conducted in Brazil May-October 2022. Physicians reported demographic/clinical characteristics and current/previous weight management. PwO reported emotional/financial impact of obesity, and completed patient-reported outcomes on HRQoL, and activity/work impairment.

**Results:**

In total, 99 physicians reported on 895 PwO. Mean ± SD PwO age was 43.1 ± 13.7, majority were female (60.9%) and white (71.7%). Mean ± SD BMI at survey was 33.8 ± 9.4 with 40.5%, 23.2% and 11.1% of PwO having class 1, 2 or 3 obesity. Weight management was most commonly at PwO request (43.4%), and consisted of prescription weight loss drug (53.6%), and dietician or physician-supervised diets (79.9% and 55.1%). Most PwO reported financial impact due to obesity treatment and reported being bothered/embarrassed by their weight. SF-36v2 physical summary scores ranged from 52.4 ± 9.3 to 45.6 ± 8.6 and mental summary scores from 45.5 ± 9.3 to 42.2 ± 12.3 (BMI < 30 to class 3 obesity). Overall work and activity impairment ranged from 20.0 ± 22.7 to 42.4 ± 28.4 (BMI < 30 and class 2 obesity) and from 24.7 ± 25.2 to 43.2 ± 32.5 (BMI < 30 to class 3 obesity), and 3.2% did not work due to obesity.

**Conclusion:**

PwO have a substantial impact on work, and financial, emotional and quality-of-life burden. Our data highlight the need for more efficacious obesity management, to help reduce work and activity impairment, improve quality of life.

## INTRODUCTION

Obesity is defined by the World Health Organization (WHO) as a chronic complex disease characterized by excessive fat deposits that can impair health (^1^). It is defined by many health organizations and medical societies as a body mass index (BMI) ≥30 kg/m^2^ and is subclassified into class 1 (30-34.9 kg/m^2^), class 2 (35-39.9 kg/m^2^) and class 3 (≥40 kg/m^2^) in adult populations (^1^-^3^). Obesity is common in Brazil, with a prevalence of 24.3% among adults in 2023, similar among women (24.8%) and men (23.8%) (^4^). Obesity rates continue to increase, with 68.1% of the Brazilian population estimated to have BMI ≥ 25 kg/m^2^ by 2030, with 29.6% having obesity (^5^). This may be associated with up to 800,000 overweight-attributable deaths in the decade up to 2030 (^6^).

Obesity has significant societal impact, with people living with obesity (PwO) in Brazil having twice the healthcare resource utilization as those with BMI of < 25 kg/m^2^ (^7^,^8^), leading to estimated direct medical costs of almost 12 billion United States dollars (USD) and almost 25 billion USD in indirect costs in 2019 (^9^). Obesity is also associated with significant costs on the individual level, with one study in the United States (US) reporting mean annual out-of-pocket costs between 2001 and 2016 were 737 USD (^10^).

However, the financial impact to individual PwO in Brazil is currently unknown. Although Brazil has a publicly funded free-at-point-of-use healthcare system, the *Sistema Único de Saúde* (Unified Health System, SUS), considerable private healthcare use remains (^11^), potentially leading to out-of-pocket costs and financial burden in PwO. Indirect costs of obesity may also be related to loss of work productivity. In a 2018 study, prior to the introduction of current treatment options, impairments in work productivity in Brazilian PwO were noted (^8^), further highlighting impact of obesity on both the individual and societal level.

Obesity also has an impact on the health-related quality of life (HRQoL) of PwO. Large cross-sectional studies in the United Kingdom (^12^) and the US (^13^) have shown the impact obesity has on the HRQoL of PwO, particularly for those with greater levels of obesity. Although a small-scale study has suggested this is the case for Brazilian PwO (^14^), to date, this has not been assessed in a national large-scale cohort.

Current Brazilian Obesity Guidelines emphasize the need for a comprehensive treatment plan, including diet, physical activity and behavioral modification (^15^). Pharmacological treatments are indicated for PwO with a previous failure to lose weight with lifestyle changes (^15^). Surgical treatment is indicated for PwO with a BMI ≥ 40 kg/m^2^, or ≥ 35 kg/m^2^ with one or more serious obesity-related complications, who have failed to lose weight with other treatment strategies, with Roux-en-Y gastric bypass surgery being the preferred method (^15^). Despite guidelines, obesity management medications (OMM) are not provided by SUS and reimbursement by private health insurance is rare, meaning OMM is commonly paid for out-of-pocket.

OMMs available on the Brazilian market at time of survey (May to October 2022) were sibutramine, orlistat, liraglutide and off-label use of semaglutide (1 mg) (^16^). Recently, the Brazilian Association for the Study of Obesity (Abeso) and Brazilian Society of Endocrinology and Metabolism (SBEM) published an expert statement, which included recently launched medications, such as higher-dose semaglutide (2.4 mg) and bupropion/naltrexone (^16^), in addition to tirzepatide, which was approved for use in obesity in June 2025 (^17^). Little is known about the implementation of these guidelines in clinical practice.

Given the relative lack of data on the management and impact of obesity in Brazil, the aim of this study was to describe the demographic and clinical characteristics of PwO in Brazil consulting with their physician for obesity management, characterize their weight loss journey, and quantify the impact of obesity on PwO finances, employment, emotional wellbeing and HRQoL. This will help identify current treatment unmet needs and challenges PwO face in Brazil.

## MATERIALS AND METHODS

### Study design and data source

This is a secondary analysis of data from the Adelphi Real World Obesity Disease Specific Programme (DSP)™, a cross-sectional survey with retrospective data analysis of PwO and their treating physicians, conducted in Brazil between May and October 2022. The DSP methodology has been previously described (^18^,^19^), validated (^20^), and demonstrated to be representative and consistent over time (^21^).

Briefly, physicians were recruited in a geographically representative manner by a local field work agency and screened for eligibility. From the eligibility survey, physicians were split into OMM prescribers or non-prescribers. They were then requested to complete a questionnaire for their next eight consecutively consulting PwO (five PwO prescribed OMM and three PwO not prescribed OMM at time of survey for physicians identified as prescribers of OMM or eight PwO not prescribed OMM for physicians identified as non-prescribers of OMM), reporting on demographic and clinical characteristics, as well as treatment patterns. Included PwO were then invited to complete a voluntary survey containing questions on the emotional impact of their obesity and a number of standardized patient-reported outcome measures (PROMs).

### Study population

Physicians were required to be a primary care provider, diabetologist, endocrinologist, or cardiologist responsible for managing at least 16 PwO per month. PwO were required to be ≥ 18 years and have a physician-confirmed obesity diagnosis (BMI ≥ 30 kg/m^2^ and/or be enrolled in a weight management program). Although all included PwO were required to have an obesity diagnosis, at data collection PwO could have achieved their weight loss goals and present with a BMI of < 30 kg/m^2^ and still receiving their weight management program. PwO participating in any clinical trial at time of survey were excluded.

### Study measures

Physicians reported on PwO demographic characteristics, which included age, biological sex, ethnicity and employment status. PwO socio-economic status was calculated from education and employment status, with low socio-economic status being defined as an educational level below having completed higher education (superior complete) and any employment status other than working full- or part-time.

Physician-reported clinical characteristics included time since diagnosis of obesity, BMI at time of diagnosis and at survey, and number and type of obesity-related comorbidities (ORCs). Physicians also reported on history of weight management, including physician types involved, reasons for starting and types of weight management, previous treatment approaches and weight loss surgery. PwO reported on the level of financial impact of obesity treatment on monthly household income, emotional impact of obesity, including levels of being bothered and embarrassed, and completed standardized PROMs related to employment and HRQoL.

### PwO-reported outcome measures

The 36-Item Short Form Health Survey version 2 (SF-36v2) was used to assess HRQoL in eight domains (general health, mental health, vitality, physical functioning, social functioning, bodily pain, limitations in role functioning due to physical health and role functioning due to emotional problems) with physical and mental component summary scores (^22^). T scores were normalized compared to US normative data (mean ± SD: 50 ± 10), with scores between 47 and 53 considered within normative range and scores < 47 as indicative of impairment (^23^).

The Work Productivity and Activity Impairment questionnaire (WPAI:SHP) was used to assess level of work and general activity impairment due to obesity, using four domains (absenteeism, presenteeism, overall work impairment and overall activity impairment, with scores ranging from 0 to 100%, with greater scores indicating greater impairment (^24^).

### Statistical analysis

All analyses were descriptive; data are presented as groups sizes with proportions, mean with standard deviation (SD) or median with interquartile range (IQR), as appropriate. PROM data are reported stratified by BMI category. Missing data was not imputed and as such the number of PwO per variable may differ. The number of PwO are reported per analysis. For some analyses, PwO were stratified based on prescription of OMM, into those being prescribed OMM at time of survey, those who were not, and those who were prescribed OMM previously but were not at time of survey.

Analyses were conducted using Stata Statistical Software 17.0 (StataCorp. 2021. College Station, TX: StataCorp LLC).

### Ethical approval

Exemption from ethical approval was granted by Pearl Institutional Review Board (protocol number #22-ADRW-136). Data collection was conducted in accordance with relevant market research and privacy regulations, including the European Pharmaceutical Marketing Research Association guidelines (^25^), US Health Insurance Portability and Accountability Act 1996 (^26^), and followed the principles of the Helsinki declaration of 1964 and subsequent revisions. PwO provided informed consent to participate.

## RESULTS

The analysis population includes 99 physicians (primary care physicians: 50.5%; diabetologists/endocrinologists: 39.4%; cardiologist: 10.1%) and 895 PwO, 379 of whom contributed self-reported data. Physicians were from the following regions: Central West (n = 2); Northeast (n = 13); Southeast (n = 58); South (n = 26).

### Demographic and clinical characteristics of PwO

Demographic characteristics of PwO are shown in **Table 1**. The mean ± SD age of PwO was 43.1 ± 13.7, and the majority were female (60.9%), White (71.7%), had never smoked (82.2%) and working full-time (69.3%). Only 3.2% of PwO were working part-time, retired or unemployed due to their obesity. The majority of PwO had completed higher education (*Ensino Superior Completo*, 59.4%) and had high socio-economic status (51.7%.)

**Table 1:** Demographic characteristics of people living with obesity

Age, years	**n = 895**
Mean ± SD	43.1 ± 13.7
Biological sex, n (%)	**n = 895**
Female	545 (60.9)
Male	347 (38.8)
Intersex	3 (0.3)
Ethnicity, n (%)	**n = 881**
White	632 (71.7)
Mixed race	150 (17.0)
Black	79 (9.0)
Asian	20 (2.3)
Smoking Status, n (%)	**n = 841**
Current smoker	46 (5.5)
Ex-smoker	104 (12.4)
Never smoked	691 (82.2)
Employment status, n (%)	**n = 895**
Working full time	620 (69.3)
Working part time	85 (9.5)
Other^a^	190 (21.2)
Working part-time / not working due to retirement / unemployed / on long term sick leave as a result of obesity?, n (%)	6 (3.2)
Education status, n (%)	**n = 374**
Illiterate / Elementary school incomplete (*Analfabeto* / *Fundamental incompleto*)	12 (3.2)
Elementary school completed / Middle school incomplete (*Fundamental I completo* / *Fundamental II incompleto*)	7 (1.9)
Middle school completed / High school incomplete (*Fundamental II completo* / *Médio incompleto*)	26 (7.0)
High school completed / higher education incomplete (*Médio completo* / *Superior incompleto*)	96 (25.7)
Higher education completed (*Superior completo*)	222 (59.4)
Other	12 (3.2)
Socio-economic status, n (%)	**n = 379**
High	196 (51.7)
Low	60 (15.8)
Indeterminate	120 (31.7)
Unknown	3 (0.8)

SD: standard deviation

a Includes retired (8.7%), homemaker (4.1%), student (3.7%), unemployed (3.0%), long-term sick leave (1.6%) and out of work due to pregnancy (0.1%)

At time of survey, PwO had been diagnosed for 2.6 ± 5.5 years (**Table 2**). Mean BMI at diagnosis was 36.0 ± 10.4 kg/m^2^, 40.1% of PwO had class 1 obesity, 27.1% class 2 obesity and 20.0% class 3 obesity. At time of survey, mean BMI was 33.8 ± 9.4, with 40.5%, 23.2% and 11.1% having class 1, 2 or 3 obesity, respectively. The median number of ORCs was 2 (IQR: 1-3), with only 20.0% of PwO having no ORCs. PwO most frequently had a single ORC (25.6% of PwO). The most commonly reported ORCs were anxiety (30.6%), hypertension and dyslipidemia (29.7% for both).

**Table 2:** Clinical characteristics of people living with obesity

Time since diagnosis, years	**n = 416**
Mean ± SD	2.6 ± 5.5
BMI at diagnosis, kg/m^2^	**n = 780**
Mean ± SD	36.0 ± 10.4
Normal weight, BMI ≥ 18.5 -< 25	4 (0.5)
Overweight (not obese), BMI ≥ 25.0 -< 30	96 (12.3)
Class 1 (low-risk obesity), BMI ≥ 30.0 -< 35.0	313 (40.1)
Class 2 (moderate-risk obesity), BMI ≥ 35.0 -< 40.0	211 (27.1)
Class 3 (high-risk obesity), BMI ≥ 40.0	156 (20.0)
BMI at time of survey, kg/m^2^	**n = 895**
Mean ± SD	33.8 ± 9.4
Normal weight, BMI ≥ 18.5 -< 25	34 (3.8)
Overweight (not obese), BMI ≥ 25.0 -< 30	192 (21.5)
Class 1, BMI ≥ 30.0 -< 35.0	362 (40.5)
Class 2, BMI ≥ 35.0 -< 40.0	208 (23.2)
Class 3, BMI ≥ 40.0	99 (11.1)
Number of ORCs at time of survey	**n = 895**
Median (IQR)	2 (1, 3)
No ORC	179 (20.0)
1 ORCs	229 (25.6)
2 ORCs	193 (21.6)
3 ORCs	124 (13.9)
4+ ORCs	170 (19.0)
Most common comorbidities ORCs at time of survey^a^, n (%)	**n = 895**
Anxiety	274 (30.6)
Hypertension	266 (29.7)
Dyslipidemia	266 (29.7)
Insulin resistance	125 (14.0)
Depression	109 (12.2)
Pre-diabetes / Impaired glucose tolerance	95 (10.6)
Type 2 diabetes mellitus	94 (10.5)
Hyperglycemia	83 (9.3)
Hypothyroidism	78 (8.7)
Non-alcoholic fatty liver disease	63 (7.0)

BMI: body mass index; ORC: obesity-related complication; SD: standard deviation

a Reported for >5% of people living with obesity

### Weight loss attempts

Weight loss attempt data was stratified by OMM prescription status, with 480 PwO being prescribed OMM at time of survey, 99 were previously prescribed OMM, but were not at time of survey, and 316 having never been prescribed OMM.

The primary physician involved in weight management of PwO at time of survey was an endocrinologist (44.3%), with primary care physicians being responsible in 41.9% of cases (**Table 3**). Endocrinologists and primary care physicians managed 50.6% and 40.8% of those receiving OMM at time of survey, 34.0% and 48.6% not receiving OMM and 47.5% and 23.7% of those who had previously received OMM, respectively.

**Table 3:** Weight loss attempts

	Overall	On OMM at time of survey	Never on OMM	Previously on OMM
Primary physician involved in weight management at time of survey, n (%)	**n = 585**	**n = 314**	**n = 212**	**n = 59**
Endocrinologist	259 (44.3)	159 (50.6)	72 (34.0)	28 (47.5)
Primary care physician	245 (41.9)	128 (40.8)	103 (48.6)	14 (23.7)
Cardiologist	50 (8.5)	17 (5.4)	23 (10.8)	10 (16.9)
Other^a^	31 (5.3)	10 (3.2)	14 (6.6)	7 (11.9)
Reason for starting most recent weight reduction program, n (%)	**n = 895**	**n = 480**	**n = 316**	**n = 99**
PwO request	388 (43.4)	232 (48.3)	110 (34.8)	46 (46.5)
Health at risk due to ORCs if weight not lost	292 (32.6)	168 (35.0)	95 (30.1)	29 (29.3)
Worsening ORCs	274 (30.6)	171 (35.6)	71 (22.5)	32 (32.3)
Treatment approaches at time of survey^b^, n (%)	**n = 895**	**n = 480**	**n = 316**	**n = 99**
Prescription weight loss drug	480 (53.6)	480 (100.0)	0 (0.0)	0 (0.0)
Dietician-supervised diet	415 (46.4)	207 (43.1)	160 (50.6)	48 (48.5)
HCP-supervised diet	381 (42.6)	194 (40.4)	140 (44.3)	47 (47.5)
HCP-agreed exercise regime	302 (33.7)	168 (35.0)	98 (31.0)	36 (36.4)
Following a low-carb diet	236 (26.4)	119 (24.8)	90 (28.5)	27 (27.3)
Previous treatment approaches^b^, n (%)	**n = 841**	**n = 450**	**n = 292**	**n = 99**
PwO’s own diet	672 (79.9)	363 (80.7)	232 (79.5)	77 (77.8)
PwO’s own exercise regime	463 (55.1)	269 (59.8)	146 (50.0)	48 (48.5)
OTC or natural remedies	299 (35.6)	171 (38.0)	105 (36.0)	23 (23.2)
Non-prescription weight loss drug	255 (26.8)	130 (28.9)	63 (21.6)	32 (32.3)
Weight loss surgeries received, n (%)	**n = 14**	**n = 6**	**n = 4**	**n = 4**
Roux-en-Y bypass	13 (92.9)	5 (83.3)	4 (100.0)	4 (100.0)
Other	1 (7.1)	1 (16.7)	0 (0.0)	0 (0.0)
Weight loss surgeries eligible for, n (%)	**n = 644**	**n = 353**	**n = 210**	**n = 81**
None	526 (81.7)	273 (77.3)	188 (89.5)	65 (80.2)
Roux-en-Y bypass	93 (14.4)	64 (18.1)	17 (8.1)	12 (14.8)
Sleeve gastrectomy	40 (6.2)	27 (7.6)	8 (3.8)	5 (6.2)
Duodenal switch with biliopancreatic diversion	6 (0.9)	5 (1.4)	1 (0.5)	0 (0.0)
Laparoscopic adjustable gastric banding	5 (0.8)	4 (1.1)	1 (0.5)	0 (0.0)
Other	2 (0.3)	1 (0.3)	1 (0.5)	0 (0.0)
Weight loss since diagnosis, %	**n = 780**	**n = 429**	**n = 263**	**n = 88**
Median (IQR)	3.6 (0.0, 10.3)	5.1(0.0, 11.2)	2.1(0.0, 9.6)	1.2(0.0, 7.9)
Weight loss since start of current attempt, %	**n = 895**	**n = 480**	**n = 316**	**n = 99**
Median (IQR)	3.2 (0.0, 9.1)	4.6 (0.0, 10.1)	2.0 (0.0, 8.2)	0.0 (0.0, 6.9)

HCP: healthcare provider; IQR: interquartile range; OMM: obesity management medication; ORC: obesity-related complication; OTC: over-the-counter; PwO: person living with obesity

a Includes nutritionist/dietician/health coach, bariatric surgeon, psychologist, diabetologist, psychiatrist, obstetrician or other healthcare provider (all <5% of total cohort)

b Those reported for >25% of total cohort listed

The most common reason for starting PwO’s most recent weight reduction program was at PwO request , ranging from 34.8% of those who did not receive OMM, to 48.3% of those receiving OMM at time of survey (**Table 3**). Other commonly reported reasons were the PwO’s health being at risk due to comorbidities if weight is not lost and worsening comorbidities (32.6% and 30.6% of overall cohort).

Overall, 46.4% of PwO had a dietitian-supervised diet (ranging from 43.1% to 50.6%) and 42.6% a healthcare provider (HCP)-supervised diet (ranging from 40.4% to 47.5%) at time of survey (**Table 3**). Other commonly reported weight loss approaches were an HCP-agreed exercise regime (33.7% of all PwO) and following a low-carb diet (26.4% of all PwO).

The majority of all PwO, regardless of OMM prescription status, had previously attempted weight loss with their own diet, ranging from 77.8% of those previously on OMM to 80.7% of those on OMM at time of survey (**Table 3**). Previously attempting weight loss through their own exercise regime was reported for 55.1% of PwO, ranging from 48.5% of those previously on OMM to 59.8% for those on OMM at time of survey. Previous use of over-the-counter or natural remedies from 23.2% of PwO previously on OMM to 38.0% of PwO on OMM at time of survey, and previous use of non-prescription weight loss drugs ranged from 21.6% of PwO not on OMM to 32.3% of PwO previously on OMM.

Physicians reported few PwO underwent weight loss surgery (n = 14, 1.6% of total cohort), the majority of whom had a Roux-en-Y bypass (92.9%, **Table 3**). Physicians reported that the majority of PwO (81.7%) were not a candidate for any type of weight loss surgery, while 14.4% were a candidate for Roux-en-Y bypass and 6.2% for a sleeve gastrectomy.

Median weight loss since diagnosis was 3.6% (IQR 0.0, 10.3%), ranging from 1.2% (0.0, 7.9%) for PwO who previously received OMM to 5.1% (0.0, 11.2%) for those receiving OMM at time of survey (**Table 3**). Median weight loss since start of the most recent weight loss attempt was 3.2% (0.0, 9.1%), ranging from 0.0% (0.0, 6.9%) for those previously prescribed OMM to 4.6% (0.0, 10.1%) for PwO receiving OMM at time of survey.

### PwO-reported financial impact of obesity

The majority of PwO reported (88.7%) not having health insurance coverage for weight loss treatment (**Table 4**). The majority of those who did (n = 35) had insurance from health insurance providers (*Operadoras de planos de saúde*). Mean PwO-reported percentage of household income spent on treatment for obesity or related conditions was 10.7 ± 13.1%, though this ranged from 3.3 ± 7.0% to 14.4 ± 13.9 for PwO previously receiving OMM and those receiving OMM at time of survey, respectively. When asked to rate impact of obesity treatment spend on their household budgets on a scale of 1 to 5, from no impact (^1^) to greatly impacted (^5^), PwO most commonly reported an impact of 1 (32.2%) or 2 (27.7%), however, 20.4% of PwO reported being an impact of 4 or 5.

**Table 4:** PwO-reported financial impact of obesity

	Overall	On OMM at time of survey	Never on OMM	Previously on OMM
Health insurance coverage for weight loss treatment, n (%)	**n = 345**	**n = 198**	**n =** ** 109**	**n = 38**
Yes	39 (11.3)	20 (10.1)	12 (11.0)	7 (18.4)
No	306 (88.7)	178 (89.9)	97 (89.0)	31(81.6)
Type of insurance coverage, n (%)	**n = 35**	**n =** ** 19**	**n = 11**	**n = 5**
Health insurance providers (*Operadoras de planos de saúde*)	29 (82.9)	17 (89.5)	9 (81.8)	3 (60.0)
Private paid out of pocket (*Particular paga* *do próprio bolso*)	6 (17.1)	2 (10.5)	2 (18.2)	2 (40.0)
Unified Health System (*Sistema Único de Saúde* – SUS)	1 (2.9)	0 (0.0)	1 (9.1)	0 (0.0)
Percentage of household income spent on treatment for obesity or related conditions	**n** ** = 191**	**n = 111**	**n = 58**	**n =** ** 22**
Mean ± SD	10.7 ± 13.1	14.4 ± 13.9	6.4 ± 10.6	3.3 ± 7.0
Impact of treatment spend on budget, n (%)	**n = 332**	**n = 196**	**n = 106**	**n = 30**
1 No impact	107 (32.2)	42 (21.4)	53 (50.0)	12 (40.0)
2	92 (27.7)	62 (31.6)	23 (21.7)	7 (23.3)
3	65 (19.6)	47 (24.0)	13 (12.3)	5 (16.7)
4	36 (10.8)	25 (12.8)	8 (7.6)	3 (10.0)
5 Greatly impacted	32 (9.6)	20 (10.2)	9 (8.5)	3 (10.0)

OMM: obesity management medication; PwO: person living with obesity; SD: standard deviation

PwO: people living with obesity

### PwO-reported emotional impact of obesity

**Figure 1:**
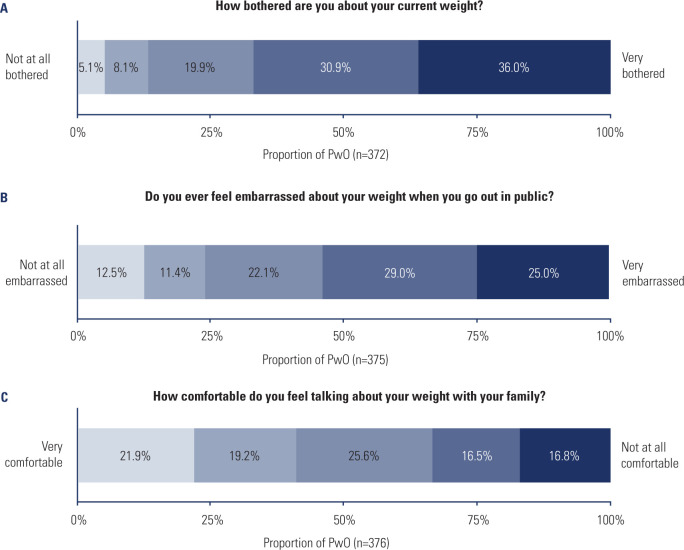
PwO-reported emotional impact of obesity. **A**) Proportions of PwO reporting being bothered by their current weight. **B**) Proportion of PwO reporting levels of embarrassment experienced by PwO when out in public. **C**) Proportion of PwO reporting levels of being comfortable discussing weight with family. PwO: people living with obesity

When asked to rate their level of being bothered about their weight on a scale of 1 to 5, from not at all bothered (^1^) to very bothered (^5^), PwO most commonly reported their level of bother at 4 (30.9%) or 5 (36.0%, **Figure 1A**). When rating their level of embarrassment about their weight when going out in public on a scale of 1 to 5, with 5 being very embarrassed, PwO commonly reported being embarrassed, replying with a 4 (29.0%) or 5 (25.0%, **Figure 1B**). In contrast, only a third of PwO reported being uncomfortable discussing their weight with their family, with 16.5% reported a 4 when asked, and 16.8% a 5 on a scale of 1 to 5, with 5 being not at all comfortable (**Figure 1C**).

### PwO-reported impact on HRQoL

**Figure 2:**
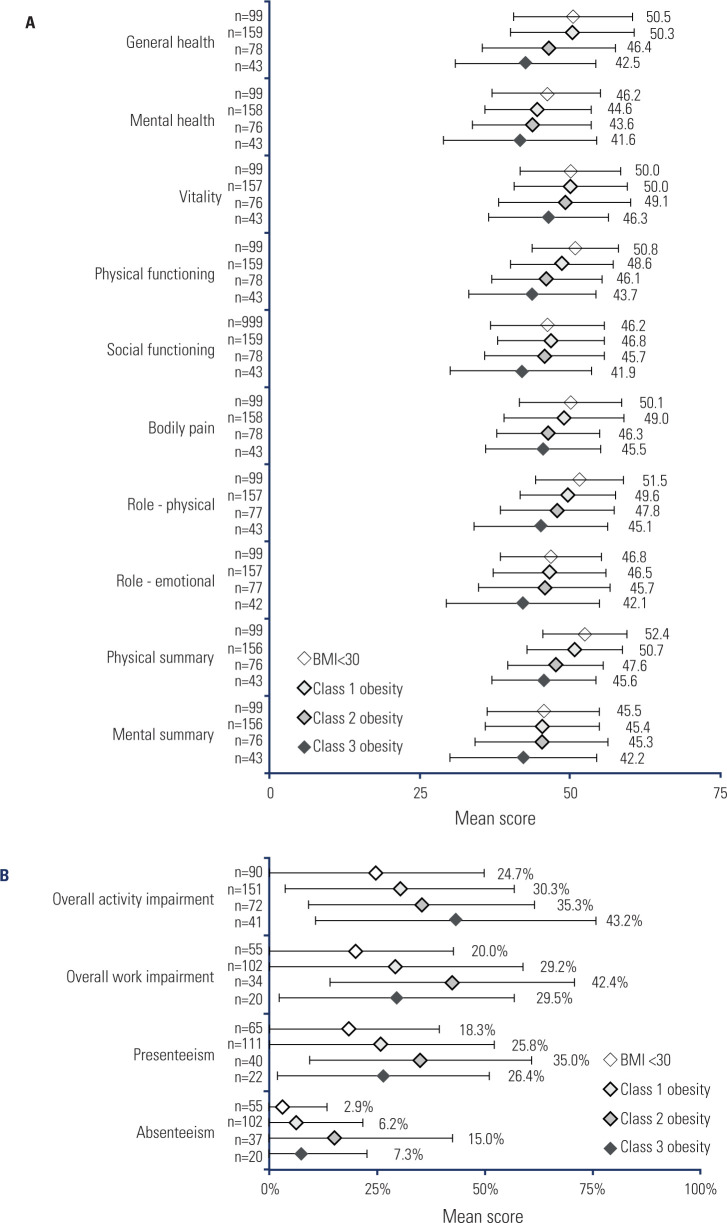
Patient-reported outcomes, split by BMI class. **A**) General health status was assessed using the SF-36v2 measure, in eight domains, with two component summary scores. Higher scores indicate better HRQoL, with scores between 47 and 53 considered within normative range and scores < 47 as indicative of impairment. **B**) Level of activity and work impairment was assessed by the WPAI, shown as percentage of impairment in each domain. Overall activity impairment covers all PwO, overall work impairment and presenteeism/absenteeism scores cover all working PwO. BMI: body mass index; HRQOL: health-related quality of life; PwO: people living with obesity; WPAI: Work Productivity and Activity Impairment

PwO reported on their HRQoL using the SF-36v2, with mean physical component summary score being 49.9 ± 8.1 and mental component summary score being 45.0 ± 10.2 (**Figure 2A**). The scores for individual health domains ranged from 44.5 ± 9.7 for mental health to 49.4 ± 9.6 for vitality in the overall sample. For PwO with BMI < 30, these scores ranged from 45.5 ± 9.3 to 50.0 ± 9.4, respectively, whereas for those with class 3 obesity, there were 41.6 ± 12.8 to 46.3 ± 10.0. Low scores in the overall sample were recorded for mental health (44.5 ± 9.7), social functioning (45.9 ± 9.7), limitations in role functioning due to emotional problems (45.9 ± 10.0) and mental component summary score (45.0 ± 10.2). For PwO with class 3 obesity, these scores were 41.6 ± 12.8, 41.9 ± 11.8, 42.1 ± 12.7 and 42.2 ± 12.3, respectively.

### PwO-reported impact of work and activities

PwO recorded a mean overall activity impairment in the WPAI, regardless of employment status, of 31.4 ± 27.4%, ranging from 24.7 ± 25.2% in those with BMI < 30 and 43.2 ± 32.5% in those with class 3 obesity (**Figure 2B**). Among employed PwO, an overall work impairment of 29.0 ± 28.3% (ranging from 20.0 ± 22.7% to 42.4 ± 28.4%). Within work impairment, PwO reported an absenteeism level of 7.0 ± 17.5% (ranging from 2.9 ± 11.4 to 15.0±27.5) and a presenteeism level of 25.3 ± 25.2% (ranging from 18.3 ± 21.0 to 35.0 ± 25.8).

## DISCUSSION

Although obesity is an increasing issue in Brazil, little data exist on the real-world management of obesity, or its impact on PwO finances, employment, emotional wellbeing and HRQoL. Here, we address this lack of data by using a large sample of consulting Brazilian PwO and their treating physicians.

Our sample represents a wide range of PwO, including substantial groups with BMI < 30 who previously had BMI > 30 and every class of obesity, ensuring that results are representative. PwO had a median number of two ORCs, with only a fifth of PwO having no ORCs. Our data suggest the presence of comorbidities may drive weight management conversations, including a need to take earlier action to decrease risk from comorbidities.

Our data make it clear that treatment is generally multifaceted, with diet or exercise interventions reported for many PwO, in addition to pharmacological or surgical treatment. The majority of PwO in our cohort were prescribed OMM (as per established quotas) at time of survey. It is important to emphasize that at the time the survey was conducted, only sibutramine, orlistat, liraglutide and low dose semaglutide were available in Brazil, and all of them are paid for by the PwO. Prescription of OMM was associated with being treated with by an endocrinologist, who were the most common treating physician for both PwO receiving OMM at time of survey and those who received OMM previously. In contrast, those PwO who had never received OMM were most commonly treated by primary care physicians, suggesting pharmacological treatment of obesity is still poorly understood and practiced by these physicians and these PwO were less likely to have been referred to specialist care for their obesity. The 2016 Brazilian Obesity Guidelines recognize endocrinologists as the specialists qualified to treat PwO (^15^). However, due to the high prevalence of obesity and overweight, it is important that other specialists and primary care physicians are trained to recognize and refer these PwO quickly, and offer pharmacological treatment to PwO.

Surgical therapy was rare in our sample, taking place for 1.6% of PwO, with most of those being a Roux-en-Y bypass. This is despite the fact that Brazil has been noted as having a high and growing rate of bariatric surgery, second only to that in the US (^27^). However, only 20.0% of PwO in our sample had class 3 obesity, making them eligible for bariatric surgery in the absence of other risk factors under Brazilian guidelines (^15^). This was also reflected in physicians’ assessment of PwO eligibility, with physicians stating that 81.7% of their consulting PwO were ineligible for any weight loss surgery. This suggests that referral for bariatric surgery in our cohort was largely according to treatment guidelines.

The majority of PwO did not have insurance coverage for their obesity care. This was reflected in the fact the majority of PwO reported spending on obesity treatment had an impact on their budget. Notably, this was the case in 78.6% of those receiving OMM at time of survey, compared to 50.0% of those who had never received OMM and 60.0% of those who had previously received OMM. These data strongly suggest a greater financial burden for those receiving OMM than for those utilizing other obesity treatment strategies. This burden may also lead to inequity of treatment, with PwO of lower socio-economic status possibly being less able to afford OMM.

Our data also clearly illustrates the mental and emotional impact of obesity. Anxiety and depression were commonly reported in our sample. The fact that obesity is strongly associated with anxiety and depression is well known (^28^,^29^), but the prevalence in our sample is noteworthy, as PwOs also showed a notable degree of emotional and mental health impact. These findings highlight the need to take PwO’s emotional and mental wellbeing into account as part of any weight management strategy. This may also be related to the stigma associated with obesity, which has been recognized as a contributing factor to reduced physical health and emotional wellbeing (^30^,^31^), as well as poorer healthcare access (^32^). Many PwO in our cohort were bothered about their weight and embarrassed about their obesity in public.

This impact on mental wellbeing was also reflected in PROMs. Taking the commonly used threshold of 47 as the upper limit of impairment in the SF-36v2 (^23^), PwO in our overall sample were impaired in the mental health, social functioning and limitations in role functioning due to emotional problem domains, as well as in the mental component summary score. This suggests obesity had a greater impact on PwO emotional aspects of their HRQoL than the physical aspects in the overall sample. This is in contrast to both a previous Brazilian study (^8^) and a meta-analysis of studies in Europe, North America and Australia (^32^), which found impacts in a range of mental and physical domains. In contrast, those with class 3 obesity scored below 47 across every domain, indicating that for those PwO with more severe obesity, both physical and mental aspects of everyday are affected.

Although our study has several strengths, including combining patient-level data reported from physicians and PwO, and the use of a large, geographically representative sample, it also has limitations. This DSP only includes those PwO who consult with their physicians, and participation of PwO may therefore not be representative of the wider PwO population, particularly due to quotas required for numbers of PwO prescribed OMM. The majority of PwO were also of high socio-economic status, like due to higher consultation frequency. Similarly, the DSP is based on a pseudo-random sample of physicians or PwO. While minimal inclusion criteria governed the selection of participating physicians, participation was influenced by their willingness to complete the survey. To minimize selection bias, physicians were asked to provide data for a consecutive series of eligible PwO. Recall bias, a common limitation of surveys, might also have affected responses of both physicians and PwO. However, physicians did have the ability to refer to medical records while completing the survey, thus minimizing the possibility of recall bias. The cross-sectional nature of this study precludes conclusions about causal relationships, and only associations can be made. Lastly, in order to facilitate comparison with other datasets, US SF-36v2 normative values were applied, which may overestimate impairments or underestimate improvements in HRQoL compared to using Brazilian normative data (^33^).

In conclusion, despite receiving weight management, consulting Brazilian PwO in our sample have a substantial comorbidity burden, which may drive physician treatment choices. PwO also have a substantial work, financial, emotional and HRQoL burden, regardless of treatment received. Our data highlight the need for better management pathways to ensure those with higher BMI have better outcomes, improved HRQoL and lessened financial burden of obesity.

## Data Availability

all data, i.e. methodology, materials, data and data analysis, that support the findings of this survey are the intellectual property of Adelphi Real World. All requests for access should be addressed directly to Lewis Harrison at lewis.harrison@omc.com.
